# Molecular mechanisms of human overgrowth and use of *omics* in its diagnostics: chances and challenges

**DOI:** 10.3389/fgene.2024.1382371

**Published:** 2024-06-04

**Authors:** Dirk Prawitt, Thomas Eggermann

**Affiliations:** ^1^ Center for Pediatrics and Adolescent Medicine, University Medical Center, Mainz, Germany; ^2^ Institute for Human Genetics and Genome Medicine, Medical Faculty, RWTH Aachen, Aachen, Germany

**Keywords:** overgrowth, omics, diagnostics, mosaicism, long read sequencing, massive parallel sequencing (MPS)

## Abstract

Overgrowth disorders comprise a group of entities with a variable phenotypic spectrum ranging from tall stature to isolated or lateralized overgrowth of body parts and or organs. Depending on the underlying physiological pathway affected by pathogenic genetic alterations, overgrowth syndromes are associated with a broad spectrum of neoplasia predisposition, (cardio) vascular and neurodevelopmental anomalies, and dysmorphisms. Pathologic overgrowth may be of prenatal or postnatal onset. It either results from an increased number of cells (intrinsic cellular hyperplasia), hypertrophy of the normal number of cells, an increase in interstitial spaces, or from a combination of all of these. The underlying molecular causes comprise a growing number of genetic alterations affecting skeletal growth and Growth-relevant signaling cascades as major effectors, and they can affect the whole body or parts of it (mosaicism). Furthermore, epigenetic modifications play a critical role in the manifestation of some overgrowth diseases. The diagnosis of overgrowth syndromes as the prerequisite of a personalized clinical management can be challenging, due to their clinical and molecular heterogeneity. Physicians should consider molecular genetic testing as a first diagnostic step in overgrowth syndromes. In particular, the urgent need for a precise diagnosis in tumor predisposition syndromes has to be taken into account as the basis for an early monitoring and therapy. With the (future) implementation of next-generation sequencing approaches and further *omic* technologies, clinical diagnoses can not only be verified, but they also confirm the clinical and molecular spectrum of overgrowth disorders, including unexpected findings and identification of atypical cases. However, the limitations of the applied assays have to be considered, for each of the disorders of interest, the spectrum of possible types of genomic variants has to be considered as they might require different methodological strategies. Additionally, the integration of artificial intelligence (AI) in diagnostic workflows significantly contribute to the phenotype-driven selection and interpretation of molecular and physiological data.

## 1 Introduction

Overgrowth (macrosomia, tall stature) is defined as a body length that is above the 97th percentile of the reference population, i.e., the individual belongs to the 3% tallest in the same age group. It has been estimated that about 5% of all children are born with a weight greater than 4,000 g (Stevenson, 2015), and that excessive birth weight represents a normal variation in some cases, as growth is at least in part influenced by family tall stature and socioeconomic status. In the recent years the term overgrowth, especially in overgrowth syndromes, has evolved to describe also disease entities with a variable phenotypic spectrum ranging from tall stature to isolated or lateralized overgrowth of body parts and or organs.

Somatic growth comprises an increase in size and volume of the body that goes along with a morphological and functional differentiation of the organ systems. It is the product of continuous and complex interactions between genes and different environmental factors (Diaz Bonilla et al., 2018).

Growth can be measured and predicted by parameters that have a normal distribution on a population level, depending on the specific intra-population and inter-population heterogeneity. The population specific physical growth is commonly evaluated on the basis of reference curves, that take into account how the growth occurs in ideal conditions.

Growth starts prenatally and usually continues until early adulthood. It is divided into four growth phases: fetal, infant, childhood and pubertal growth.

Prenatal growth is routinely followed with serial ultrasonography (depicting changes of biparietal diameter, abdominal circumference, and femur length) (Guihard-Costa et al., 1991). Prenatal overgrowth can be observed either in the fetal phase, or it presents as either macrosomic neonates (with an estimated fetal weight or birth weight greater than 4000 g), or with length and weight ≥97th centile. These children are called large for gestational age (LGA) when they have a generalized overgrowth (Manor and Lalani, 2020). Anthropometric postnatal indicators usually used for reference growth curves are height and weight for age, body mass index (BMI), and head circumference (Alves Junior et al., 2023).

Pathologic overgrowth may be of prenatal or postnatal onset. It either results from an increased number of cells (intrinsic cellular hyperplasia), hypertrophy of the normal number of cells, an increase in interstitial spaces, or from a combination of all of these (Stevenson, 2015).

## 2 Overgrowth syndromes

Overgrowth syndromes belong the growing number of rare diseases (Table 1). The incidences of the most common of these syndromes, Beckwith-Wiedemann-syndrome spectrum (BWSp) and Sotos syndrome (SOTOS), has been estimated around 1:12,000 to 1:14,000, whereas the other syndromes are extremely rare. However, due to the variable clinical penetrance over these disorders, they are probably underdiagnosed.

**TABLE 1:** Overview on overgrowth syndromes with known monogenetic causes (This list might not be complete, tall stature syndromes due to connective tissue disturbances (e.g., Marfan syndrome) are not listed. The syndromes are listed in alphabetical order). (Putative) functional interactions between the underlying genes are shown in Figure 1. (*IC1, IC2: in BWSp, molecular disturbances affect inprinting control regions (ICs) with a consequence for expression of imprinted genes clustering in 11p15.5, including *CDKN1C*. For this gene, pathogenic variants causing BWSp have been described. ° Pathogenic variants in *PIK3CA* and *PTEN* are associated with allelic disorder, like Cowden syndrome (CS), Congenital Lipomatous Overgrowth of the trunk with lymphatic, capillary, venous, and combined-type Vascular malformations, Epidermal naevi, Scoliosis/Skeletal and spinal anomalies (CLOVES), Bannayan-Riley-Ruvalcaba syndrome (BRRS), PTEN Hamartoma Tumor Syndrome (PHTS) and *PTEN-*related Proteus-like syndrome (PLS). NR not yet reported).

Syndrome	Acronym	OMIM	Genomic loci	Chromosome	Role in chromatin modification	Neoplasia	(Cardio) vascular anomalies	Neurodevelopmental features	genereviews
Beckwith-Wiedemann syndrome spectrum	BWSp	130650	IC2*, IC2*, CDKN1C	11p15.5		yes	yes	no	Shuman et al. (1993)
Cantu syndrome		239850	*ABCC9*	12p12.1		NR	yes	yes	Grange et al. (1993)
Cohen-Gibson syndrome	COGIS	617561	*EED*	11q14.2	yes	NR	yes	NR	Sequerra Amram Cohen et al. (1993)
Gobal developmental delay, lung cysts, overgrowth, and Wilms tumor	GLOW	618272	*DICER1*	14q32.13		yes	NR	yes	Schultz et al. (1993)
Houge-Janssens syndrome 1	HJS1	616355	*PPP2R5D*	6p21.1		NR	NR	yes	no
Hypoinsulinemic hypoglycemia with hemihypertrophy	HIHGHH	240900	*AKT2*	19q13.2		NR	NR	NR	no
Imagawa-Matsumoto syndrome	IMMAS	618786	*SUZ12*	17q11.2	yes	NR	NR	yes	no
Intellectual developmental disorder with autism and macrocephaly	IDDAM/AUTS18	615032	*CHD8*	14q11.2	yes	NR	NR	yes	Mitchel et al. (1993)
Klippel-Trenaunay-syndrome	KTS	149000	unknown	8q22.3		no	yes	NR	no
Kosaki overgrowth syndrome	KOGS	616592	*PDGFRB*	5q32		NR	NR	yes	no
Lui-Jee-Baron syndrome	LJBS	301114	*SPIN4*	Xq11.1	yes	NR	NR	NR	no
Luscan-Lumish syndrome	LLS	616831	*SETD2*	3p21.31	yes	NR	NR	yes	Pappas et al. (1993)
Macrocephaly, dysmorphic features and psychomotor retardation	MDFPMR	617011	*HERC1*	15q22.31		NR	NR	yes	no
Macrocephaly, neurodevelopmental delay, lymphoid hyperplasia, and persistent fetal hemoglobin	MNDLFH	619769	*ZBTB7A*	19p13.3		NR	yes	yes	no
Malan syndrome	MALNS	614753	*NFIX*	19p13.13	yes	NR	yes	yes	no
Parkes-Weber syndrome/Capillary malformation-arteriovenous malformation	PKWS/CMAVM	608354	*RASA1*	5q14.3		NR	yes	NR	Bayrak-Toydemir et al. (1993)
Perlman syndrome	PRLMNS	267000	*DIS3L2*	2q37.1		NR	yes	yes	no
PIK3CA-related overgrowth spectrum°	PROS	e.g., 612918, 213089	*PIK3CA*	3q26.32		yes	yes	no	Mirzaa et al. (1993)
	CS	615108							
	CLOVES	612918							
*Proteus* syndrome	PS	176920	*AKT1*	14q32.23		yes	yes	No	Biesecker et al. (1993)
PTEN Hamartoma tumor syndromes^	PHTS	158350	*PTEN*	10q23.31		yes	yes	yes	Yehia et al. (1993)
	CS	158350							
	PLS	158350							
	BRRS	158350							
Rahman syndrome	RMNS	617537	*HIST1H1E*	6p22.2	yes	NR	NR	yes	Burkardt et al. (1993)
Simpson-Golabi-Behmel syndrome type 1	SGBS1	312870	*GPC3*	Xq26.2		yes	yes	yes	Klein et al. (1993)
Smith-Kingsmore syndrome	SKS	616638	*MTOR*	1p36.22		NR	yes	yes	no
Sotos syndrome	SOTOS	117550	*NSD1*	5q35.3	yes	yes	yes	yes	Tatton-Brown et al. (1993a)
Tatton-Brown-Rahman syndrome	TBRS	615879	*DNMT3A*	2p23.3	yes	yes	in discussion	yes	Ostrowski et al. (1993)
Tenorio syndrome	TNORS	616260	*RNF125*	18q12.1		NR	yes	yes	no
Thauvin-Robinet-Faivre syndrome	TROFAS	617107	*FIBP*	11q13.1		yes	yes	yes	no
Weaver syndrome	WVS	277590	*EZH2*	7q36.1	yes	yes	yes	yes	Tatton-Brown et al. (1993b)

Though for the majority of these monogenetic disorders an autosomal-dominant mode of inheritance has been suggested, many overgrowth cases occur postzygotically. Accordingly, the patients show somatic mosaicism and an asymmetric distribution of features. Some of the syndromes only exhibit confined overgrowth of one or a few regions of the body (e.g., a single organ, extremity, body-side (hemihyperplasia), entire head (macrocephaly)).

Depending on the underlying physiological pathway affected by pathogenic genetic alterations, overgrowth syndromes are associated with a broad spectrum of neoplasia predisposition, (cardio)vascular and neurodevelopmental anomalies, and dysmorphisms (Table 1).

In fact, each monogenic macrosomia syndrome is defined by a set of clinical features, but their clinical variability is considerable, and the features often overlap. Therefore, the clinical diagnosis of overgrowth disorders is often impeded. As a consequence, the decision about genetic testing is difficult as well, and reports on the increasing application of high throughput assays in diagnostics of these entities illustrate their genetic heterogeneity and the need to apply massive parallel sequencing (MPS) approaches (Brioude et al., 2019). Additionally, these deep sequencing assays also harbor the potential to address and identify mosaic constitutions (Shen et al., 2020; Mussa et al., 2022) (see below).

The precise molecular diagnosis is necessary as the basis for an appropriate clinical management, including monitoring, therapy and genetic counselling of the patients and their families. In addition to the disease-specific health issues, comorbidities and social seclusion due to disfigurement and impaired mobility are major challenges for the patients.

## 3 Cellular and physiological bases of overgrowth

### 3.1 Skeletal growth

In the bone the proliferating zone contains flattened, stacked chondrocytes that are mitotically active, proliferate and form columns. The chondrocytes at the lower end of this zone will begin to swell due to increased synthesis of intracellular organelles and cytoplasmic water intake, becoming hypertrophic chondrocytes (HCCs) that form the hypertrophic zone. At the lower end of this zone some of the HCCs will become apoptotic, while others differentiate into osteoblasts that form the primary spongiosa (Tsang et al., 2015).

The major molecular effectors for the skeletal growth consequently are essential for the functional aspects of the different zones in the growing bone from the proliferating-to the osteoblast-zone.

**FIGURE 1:**
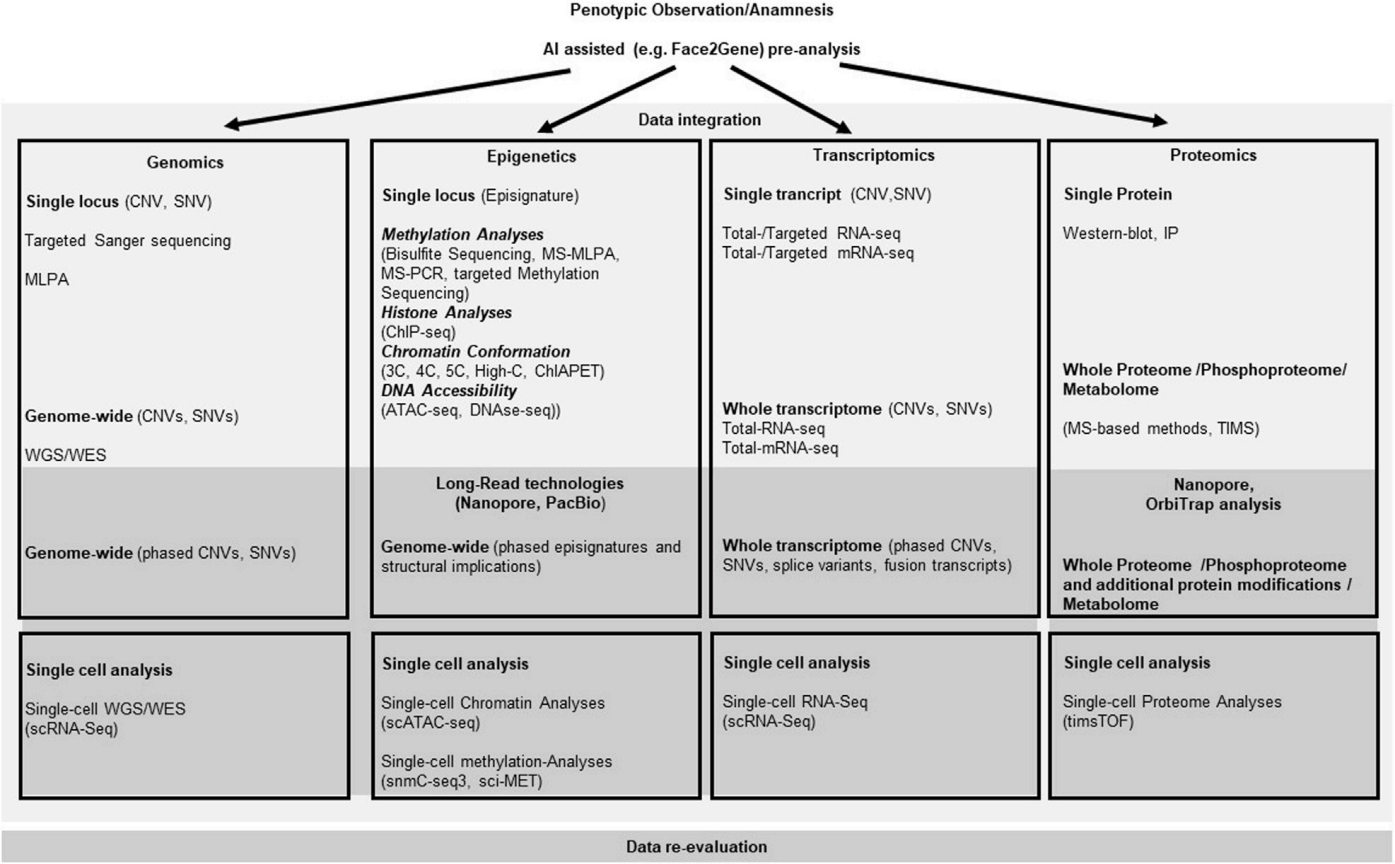
Molecular pathways for somatic growth and causative effectors for overgrowth entities. The cellular localization of the relevant gene products is roughly presented relative to the nuclear- and outer cell-membranes. Arrows depict activating, bars illustrate blocking signaling effects between two molecules. Kinked arrows illustrate transcription of a depicted gene. Circles illustrate functional multiprotein complexes. Overgrowth syndromes are listed with their abbreviations at the sites of their causative effect (i.e., mutations in the gene linked to the disease). (”?” indicates that the effect between two molecules has not been functionally described yet. For disease abbreviations see Table 1).

In the resting zone and the early proliferative chondrocytes parathyroid hormone-related protein (PTHrP) is produced, that represses Indian Hedgehog (IHH). IHH binds as a ligand to the extracellular domain of the PTCH receptor and relieves the repressive effects on the G protein-coupled receptor Smoothened (SMO). SMO then inhibits the proteolytic cleavage of the Zinc finger protein GLI (glioma-associated oncogene), a transcriptional activator that then is translocated into the nucleus and promotes the transcription of IHH/GLI target genes such as *RUNX2, PTHrp* or *SOX9* (Figure 1). Proliferating chondrocytes express FGFR3 that downregulates *IHH* expression and inhibits the proliferation (via the JAK–STAT1 pathway), thus determining the length of chondrocyte columns in the proliferating zone. Signaling through FGFR3 in growth plate chondrocytes activates STAT1, ERK1/2, and p38 intracellular signals and downregulates AKT (reviewed in: (Ornitz and Marie, 2015)). PTHrP forms a concentration gradient along the bone that ensures the transcription of *IHH* in the hypertrophic zone, when the source of PTHrP is sufficiently distant, thus establishing a negative PTHrP-IHH feedback loop that keeps proliferating chondrocytes in the proliferative pool, thus maintaining and organizing the activity of the growth plate. IHH stimulates chondrocyte proliferation directly and stimulates the distant PTHrP synthesis. By doing so IHH expression determines the distance from the resting zone and the end of the hypertrophic zone, where chondrocytes stop proliferating and undergo hypertrophic differentiation to form the bone collar (Kronenberg, 2003). Glypican 3 (GPC3) is a heparan sulfate glycoprotein on the surface of cell membranes. GPC3 inhibits IHH signaling by binding to this growth factor at the cell surface and inducing its endocytosis and degradation, as suggested by data generated in Gpc3-null mice. Loss-of-function variants in *GPC3* cause the Simpson-Golabi-Behmel syndrome type 1 (SGBS), at least in part, due to increase of IHH signaling (Capurro et al., 2009). In addition it was shown that GPC3 binds specifically through its N-terminal proline-rich region to both Insulin-like growth factor IGF-2 and IGF-1R, thus stimulating the phosphorylation of IGF-1R and the downstream signaling in an IGF-2-dependent way (Cheng et al., 2008).

Hypertrophic chondrocytes stop proliferating, enlarge (hypertrophy) and change their genetic program to synthesize collagen 10 (ColX), as well as a number of other gene-products that regulate swelling, hypertrophy, cellular metabolism and apoptosis. The most important factors are: Growth-hormone (GH), Insulin-like-growth-factor-1 (IGF-1), Hypoxia inducible factor 1-alpha (HIF1-α), sex determining region Y-box 9 (SOX9), fibroblast growth factor receptor 1 (FGFR1), bone morphogenetic protein 2 (BMP2), receptor activator of nuclear factor kappa-B ligand (RANKL), vascular endothelial growth factor (VEGF) and matrix metallopeptidases-9/13 (MMP-9/13) (Hallett et al., 2021).

GH and IGF-1 are direct regulators of chondrocyte hypertrophy. HIF1-α is a transcription factor amongst others for genes involved in glucose transport and serves as a survival factor by elevating expression of *SOX9* and glycolytic enzymes (Semenza, 2000). *NSD1*, that is altered in Sotos syndrome, can directly activate the expression of *HIF1α*, thus affecting the chondrogenic differentiation through its regulation of Sox9 expression (Shao et al., 2021). SOX9 activates Col10a1 transcription in hypertrophic chondrocytes by binding to its promoter, assuring chondrocyte hypertrophy (Shao et al., 2021). ColX is a short chain collagen that forms aggregates in the territorial matrix of hypertrophic chondrocytes (Shen, 2005). *FGFR1* is prominently expressed in hypertrophic chondrocytes and overlaps with *FGFR3*-expression in prehypertrophic chondrocytes. FGFR1 signaling seems to be important for the limitation of the hypertrophic chondrocyte zone most likely via the downstream signaling pathways, including Ras/Raf-MEK-MAPKs (mitogen-activated protein kinases), phosphatidylinositol-3 kinase/protein kinase B (PI3K/AKT) as well as the signal transducer and activator of transcription (STAT) (reviewed in: (Ornitz and Marie, 2015)). FGFR1 signaling can be modified by intracellular FGF1-binding protein FIBP. Pathogenic homozygous variants of FIBP cause the Thauvin-Robinet-Faivre syndrome (TROFAS) (Duzenli et al., 2023). Another modulator of the Ras-Raf signaling is the E3-ubiquitin ligase HERC1. Deficiency for HERC1 in the macrocephaly, dysmorphic facies, and psychomotor retardation syndrome (MDFPMR) increases the levels of C-RAF and of phosphorylated ERK (Pedrazza et al., 2023). Other growth-factor receptors also have influence on the Ras/Raf-MEK-MAPKs. The *DGFRB* gene encodes platelet-derived growth factor receptor-beta, a cell surface tyrosine kinase that attenuates Ras/PI3K activity. Pathogenic variants in *DGFRB* cause Kosaki overgrowth syndrome (KOGS) (Takenouchi et al., 2019). BMP2 expression is essential for the hypertrophic state of the chondrocytes by delaying the terminal differentiation of hypertrophic chondrocytes. It results in an increase in IHH and Col10a1 expression in the pre-hypertrophic and hypertrophic zones as well as in *RANKL* expression (Valcourt et al., 1999). RANKL molecules on osteoblasts work as receptors to stimulate osteoblast differentiation. The Runt-related transcription factor-2 (Runx2) supports the hypertrophy and regulates transdifferentiation of chondrocytes into osteoblasts (Qin et al., 2020). Runx2 transcriptionally regulates genes critical for extracellular matrix ECM synthesis, like the matrix metalloproteinase 13 (MMP13) (Inada et al., 2004), thus limiting the expansion of the hypertrophic zone as well as vascular invasion of the ossification center via VEGF (Zelzer et al., 2001).

DIS3-Like 3-prime-5-prime Exoribonuclease 2 (DIS3L2), in which pathogenic variants have been linked to Perlman syndrome (PRLMNS), is an RNA-binding protein with 3′-5′ exoribonuclease activity. DIS3L2 is predominantly localized in the cytoplasm. It can recognize, bind, and mediate the degradation of cytoplasmic uridylated RNA, including pre-/mature microRNAs, mRNAs, and some other non-coding RNAs. The precise mechanism leading to the associated overgrowth remains to be solved, but one can speculate that it is caused by the toxic accumulation of defective RNA species and/or by loss of specific RNA(s) that require DIS3L2 activity for function in the molecular cascades relevant for growth (Menezes et al., 2018).

### 3.2 Growth-relevant signaling cascades

The major effectors for somatic growth during the prenatal phase are the insulin and insulin-like growth factors (INS, IGF-1, IGF-2). They can bind and activate the type 1 insulin-like growth factor receptor (IGF1R), a class II receptor tyrosine kinase (RTK), that plays a critical role in cell growth and differentiation by activating the PI3K-AKT-mTOR and RAS-RAF-mitogen-activated protein kinase (MAPK) pathways (Chitnis et al., 2008). Parkes-Weber syndrome (PKWS) is caused by pathogenic variants in the RAS p21 protein activator 1 (RASA1), a Ras-GTPase activating protein. The RAS-pathway controls transcription of growth and differentiation relevant genes and is activated by IGF-1R signaling via the Raf-MEK-ERK signaling. Pathogenic variants in *RASA1* lead to an increased activation of MEK-ERK that increases cell size and initiates arteriovenous malformations (AVM) (Greysson-Wong et al., 2023). Another modifier of the MAPK-pathways is the E3 ubiquitin ligase RNF125. Pathogenic variants in *RNF125* putatively influence the MAPK–pathways with an yet unclear mechanism and cause the Tenorio syndrome (TNORS) (Tenorio-Castano et al., 2021). The protein phosphatase-2A (PP2A), an intracellular serine/threonine phosphatase regulates the acitivity of the MAPK-pathways and can cause Houge-Janssens syndrome-1 (HJS1) (Douzgou et al., 1993). A schematic overview of the macrosomia causing signaling cascades are depicted in Figure 1.

Several molecular alterations can be causative for BWSp associated overgrowth. These are *IGF2*-overexpression, *H19* reduced expression (observed often in concert) or loss-of-function of the Cyclin-Dependent Kinase Inhibitor 1C (CDKN1C). IGF-2 binds to several IGF/insulin (INS) receptors (IGF-1R, INSR, IGF-1/INSR hybrids and IGF-2R) to exert autocrine, paracrine and endocrine effects (Sferruzzi-Perri et al., 2017). Overexpression of *IGF2* significantly increases the weights of mice at the age of 5–10 weeks and significantly affects the adult glycolipid metabolism, most likely due to changes in the IGF-2-IGF-1R-STAT3/AKT2-GLUT2/GLUT4 pathway. Due to different effects of *IGF2* overexpression on tissues involved in glycolipid production, storage or consumption, the specific process by which *IGF2* overexpression regulates its downstream pathway in each tissue is not completely understood (Zhang et al., 2023). The long-noncoding RNA *H19* is a primary microRNA precursor for miR-675 (Cai and Cullen, 2007), The miR-675-3p directly inhibits the expression of *IGF1R* (Wang et al., 2018). Consequently, reduction or loss of *H19* expression increases the available IGF1R signaling. CDKN1C is a member of the Cip/Kip family of cell cycle inhibitors, which restrict the eukaryotic cell cycle at G1, thus negatively controlling cellular proliferation. In addition it directly modifies the AP-1/c-Jun-activity through direct binding to these transcription factors after binding of unspecified activator- or inhibitor-factors (Kullmann et al., 2021). *C-Jun* is one transcriptional target of IGF-1R signaling through the Ras-JNK pathway and increasing the activity of c-Jun due to a loss-of-function variant in *CDKN1C* suggestively could equal an enhanced transcription due to enhanced IGF1R signaling.

Overactivation of the IGFR-I pathways can also be triggered by increased maternal nutrition or placental function, resulting in macrosomia. In neonates born to mothers with maternal hyperglycemia (because of obesity or diabetes), it is suggested that an intrinsic fetal pancreatic beta-cell hyperplasia with resulting fetal hyperinsulinism increases the glucose transport across the placenta, thus assisting the glycemic control of the mother, but also increasing fetal size and alpha-glycerophosphate synthesis in fetal adipocytes, supporting a further increase in fetal adiposity. However the macronutrient metabolism cannot completely explain the relationship between maternal metabolic conditions and macrosomia, because lifestyle modification or maternal insulin adjustment does not always reduce the incidence of macrosomia (Nahavandi et al., 2019). Gain-of-function variants in *ABCC9*, which encodes an ATP-sensitive potassium channel subunit relevant also for insulin secretion and thus blood glucose homeostatis, cause Cantu syndrome (McClenaghan and Nichols, 2022). Other maternal and placental factors that affect the supply and uptake of nutrients to the fetus can also contribute to fetal overgrowth. These include physical activity, uteroplacental blood flow, placental transfer and genetic components (i.e., ethnicity) (Damhuis et al., 2021).

### 3.3 Asymmetric/mosaicism

Some of the overgrowth syndromes present with confined overgrowth of one or a few regions of the body, e.g., BWSp, *PTEN*- and *PIK3CA*-associated disorders (PHTS, PROS), *Proteus* syndrome (PS), Parkes-Weber syndrome (PKWS), CLOVES (Congenital Lipomatous asymmetric Overgrowth of the trunk with lymphatic, capillary, venous, and combined-type Vascular malformations, Epidermal naevi, Scoliosis/Skeletal and spinal anomalies Syndrome) and Klippel-Trenaunay-syndrome (KTS). Their segmental or mosaic overgrowth can be explained by causative pathogenic variants that occur after the formation of the zygote, resulting in the presence of the molecular defects in only a subset of cells (i.e., a mosaic distribution of the variant). Such variants are often overactivating variants of the phosphoinositide-3-kinase/protein kinase B/mammalian target of rapamycin pathway (PI3K/AKT/mTOR) (pathogenic variants in *PIK3CA* in PROS, KTS; mutations of *AKT1* in PS, of *AKT2* in hypoinsulinemic hypoglycemia with hemihypertrophy (HIHGHH), of mTOR in Smith-Kingsmore syndrome (SKS)) or deactivating variants in repressors of this pathway (the phosphatase and tensin homolog (*PTEN*) in PHTS and PS) (Yehia et al., 1993).

Pre- and Postnatal human body growth is mainly determined by the growth of the long bones of the axial skeleton (vertebrae and ribs) and the appendicular skeleton (limbs). The skeleton is made of two tissues (cartilage and bone) and consists of 3 cell types (chondrocytes, osteoblasts and osteoclasts) (Karsenty, 2003). Endochondral ossification begins with primary ossification centers in the cartilage during fetal development approximately between weeks 8 and 12 of gestation, followed by mineralization of bone starting in the third trimester. Growth plates in the femurs form from secondary ossification centers around gestational week 34. From this time point linear growth continues until the end of puberty, when the growth plates fuse (Kovacs, 2014). The growth plates consist of a resting zone which contains small mitotically inert round chondrocytes (stem-like or progenitor cells). They require the activity of ß-catenin for cell maintenance (Candela et al., 2014). *NSD1* encodes a transcriptional coregulator protein that epigenetically increases transcription of WNT10B, that in turn increases Wnt/β-catenin signaling (Zhang et al., 2022). *NSD1* loss-of-function variants that cause SOTOS (Tatton-Brown et al., 1993a) thus can push cells of the resting zone to proliferate, consequently increasing the bone length. It can also act via a different cascade involving SOX9, which is discussed below.

Malan syndrome (MALNS) is an overgrowth syndrome that shares characteristics with SOTOS. It is not caused by pathogenic variants in *NSD1*, but heterozygous variants or deletions of the *NFIX* gene. How the *NFIX* product leads to this overgrowth phenotype is still unclear. One explanation could be the interaction of NFI proteins with Sloan‐Kettering oncogene (SKI) (Baldwin et al., 2004), that suppresses SMAD‐dependent TGF‐β signaling (Muench et al., 2018).

TGF-β1 influences already the migration of cells to the site of future skeletogenesis and the formation of cellular condensations, thus directing the general shape of the future skeletal elements (Kanaan and Kanaan, 2006). The TGF-β1 pathway and the canonical WNT/β-catenin pathway stimulate each other through the Smad pathway, and also through phosphatidylinositol 3-kinase/serine/threonine kinase signaling (PI3K/Akt) (Vallee et al., 2017). It is therefore suggestive that pathogenic variants in *NFIX* lead to a blocking of SKI, that in turn increases the existing SMAD‐dependent TGF‐β signaling, similar to the effect of pathogenic *NSD1* variants.

### 3.4 Episignature/chromatin modification

The recent years have shown that epigenetic modifications (i.e., DNA methylation, histone modification) play a critical role in the manifestation of rare diseases (Martinez-Delgado and Barrero, 2022). For more than 60 congenital neurodevelopmental disorders, specific episignatures have been reported, among them overgrowth disorders (Table 1).

Like *NSD1*, the *enhancer of zeste homolog 2* (EZH2), is a histone methyltransferase and presents the catalytic subunit of the polycomb repressive complex 2 (PRC2), that is required for initial targeting of genomic region to be epigenetically silenced by catalyzing histone H3 lysine 27 tri-methylation (H3K27me3) (Montgomery et al., 2005). EZH2 activity is inhibited by AKT through the phosphorylation of serine 21, which blocks binding of EZH2 to histone 3 (Cha et al., 2005). EZH2 colocalizes with and regulates beta-catenin targeted genes across the genome (Bracken et al., 2006). Heterozygous *EZH2* loss of function variants cause Weaver syndrome (WVS), according to current data, most likely due to PRC2 defects with consequently altered WNT/beta-catenin signaling in stem cells that result in overgrowth of the skeleton, visceral organs and/or the brain (Lui and Baron, 2023). Other components of the PRC2 complex are EED and SUZ12. Pathogenic variants in these genes can also result in overgrowth due to loss of PRC2 functionality similar to EZH2 in Weaver syndrome. Pathogenic *SUZ12* variants are causative for the Imagawa-Matsumoto syndrome (IMMAS) and pathogenic variants in *EED* cause the Cohen Gibson Syndrome (COGIS) (Cyrus et al., 2019).

Another methyltransferase associated with overgrowth is the DNA de-novo methyltransferase 3A (DNMT3A). Pathogenic variants in *DNMT3A* cause Tatton-Brown-Rahman syndrome by focal, canonical hypomethylation of genomic DNA (Smith et al., 2021).

Further effectors that influence epigenetic marks or mediate epigenetic effects like *CHD8, HIST1H1E, SETD2, SPIN4, ZBTB7* and *DICER1* can also cause complex overgrowth phenotypes when mutated (Figure 1; Table 1).

## 4 Diagnosis of overgrowth syndromes

The diagnosis of overgrowth syndromes as the prerequisite of a personalized clinical management can be challenging, due to their clinical and molecular heterogeneity. Physicians should consider molecular genetic testing as a first diagnostic step in overgrowth syndromes. In particular, the urgent need for a precise diagnosis in tumor predisposition syndromes has to be taken into account as the basis for an early monitoring and therapy.

**FIGURE 2:**
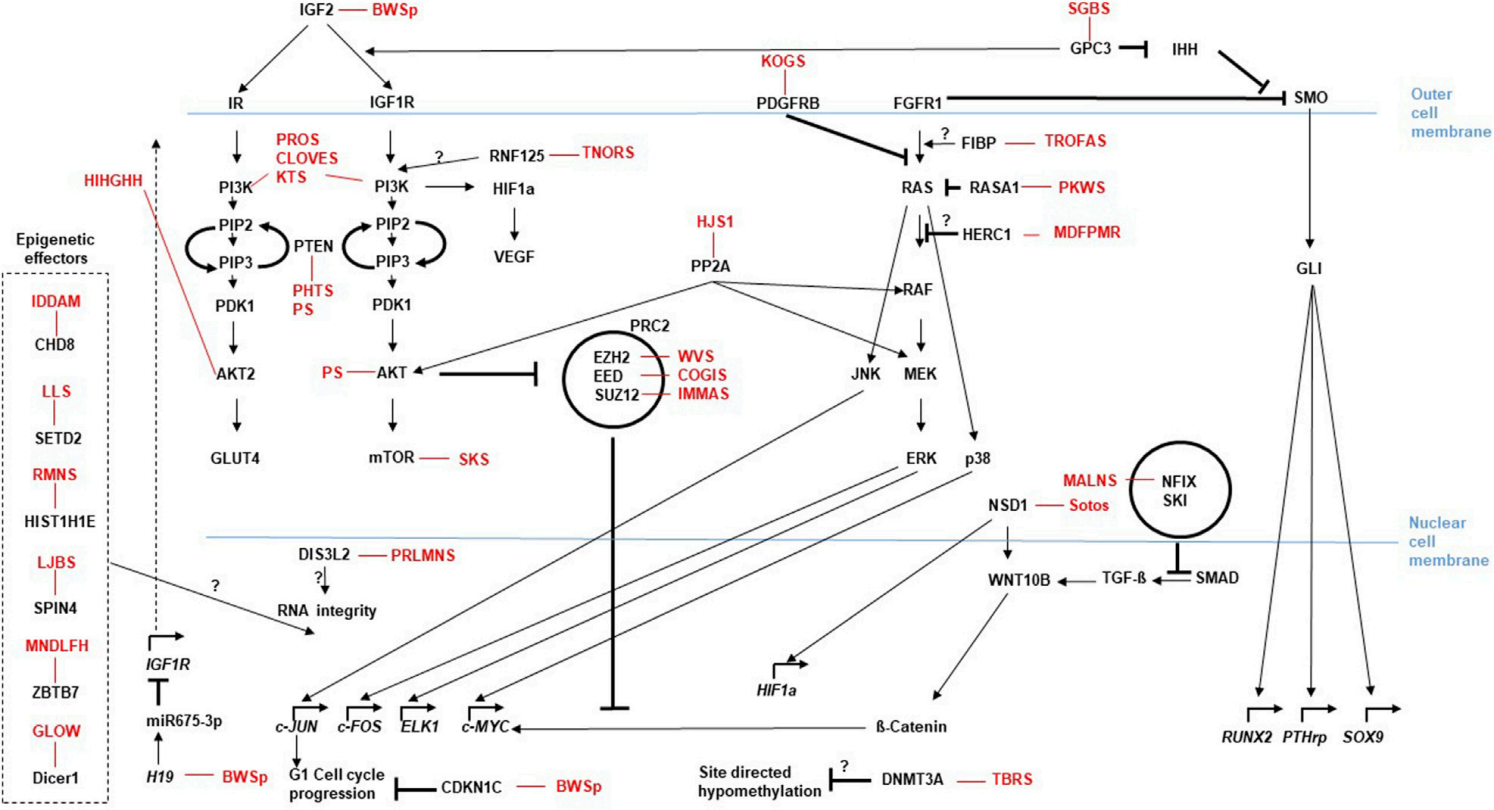
Current and emerging Omics techniques and their usage in the diagnostic flow-chart for rare overgrowth syndromes. The diagram depicts the different omics fields (genomics, epigenetics, transcriptomics and proteomics) currently used to address diagnostic aspects of overgrowth entities that follow the medical anamnesis. Selection of the most promising diagnostic approach can be assisted by AI based techniques like Face2Gene (compare main text). Methods listed are not complete, but focus on the most prominent ones.

With the (future) implementation of massive parallel sequencing (MPS) approaches and further *omic* technologies (Figure 2), clinical diagnoses can not only been verified, but they also confirm the clinical and molecular spectrum of monogenic disorders, including unexpected findings and identification of atypical cases. Additionally, the integration of artificial intelligence (AI) in diagnostic workflows significantly contribute to the phenotype-driven interpretation of molecular and physiological data. Currently, the routine clinical, biochemical and molecular testing approaches and the interpretation of their results often run in parallel, but future approaches require the comprehensive AI-based integration of these datasets (Smirnov et al., 2023).

### 4.1 Clinical diagnosis

The clinical phenotypes of overgrowth syndromes can be subtle and show significant overlapping features, thereby complicating the clinical diagnosis and a reliable estimation of syndrome associated risks.

In syndromes mainly caused by somatic pathogenic variants the phenotypes might evolve progressively (e.g., PS), and key-symptoms might not be present in early stages. As these syndromes can be associated with a high risk of neoplasia, an early diagnosis is required to start clinical treatment as early as possible. Due to the difficulties of clinical diagnoses, a comprehensive diagnostic workup should be considered as early as possible. The implementation of comprehensive molecular diagnostic approaches in the last years has not only contributed to the identification of new pathogenic variants in the known overgrowth syndromes, but also has led to the identification of new diseases and has expanded the knowledge about overlapping phenotypes.

Recognition of rare monogenetic syndromes and their atypical appearances depends on the experience of the examining physician. However, a careful and comprehensive documentation of the clinical features over time, as well as anamnesis of the family history are the prerequisite for the interpretation of molecular data in the course of the diagnostic workup.

Standardization of phenotyping can be achieved by application of Human Phenotype Ontology (HPO) terms, a standardized vocabulary of phenotypic features (Kohler et al., 2017). In the era of diagnostic whole exome and whole genome sequencing (WES, WGS), the use of HPO terms join clinical information with computational tools and resources, thereby allowing the interpretation of WES/WGS datasets in respect to their clinical significance. The data-driven identification of disease-causing variants is achieved by the application of a broad and growing set of bioinformatic tools like Phenomizer (Kohler et al., 2009) which help the molecular biologists and clinicians to filter genetic variants.

With the development in computational algorithms these approaches have been significantly refined, incorporating also AI-assisted analyses. This is used by computational programs like Face2Gene with its algorithm DeepGestalt (Gurovich et al., 2019), enabling a clinical diagnosis based on AI-assisted facial image analysis. These computational approaches are increasingly used to prioritize molecular analyses. So does the “Prioritization of Exome Data by Image Analysis” (PEDIA) workflow integrate portrait photos directly into the interpretation of sequence variants (Hsieh et al., 2019). One can well imagine that such approaches will become more relevant in the future.

### 4.2 Biochemical diagnosis

Overgrowth syndromes present with overlapping phenotypes and due to the affected signaling pathways often have converging biochemical alterations. Therefore, biochemical analyses only represent an auxiliary evaluation test in addition to growth velocity analyses, karyotype studies and bone age. The focus of biochemical parameters lies on IGF-1, IGFBP-3, free T4, and TSH (Manor and Lalani, 2020). As for the overgrowth syndromes discussed above only few show syndrome-specific biochemical alterations. Analyses in Silver-Russell-syndrome (SRS, a short stature syndrome) patients, that have causative opposing molecular alterations to the BWSp, suggest that IGF-2 serum levels during childhood are unlikely to reflect the methylation status at the *IGF2* locus. (Binder et al., 2006), although serum IGF-2 in combination with IGF-1 and IGFBP-3 can add to the clinical signs of SRS patients and help to perform targeted genetic testing (Binder et al., 2017). A Chilean study analyzed cord sera obtained from term newborns exhibiting various patterns of intrauterine growth. The sera were assayed for IGF-1, IGF-2, IGFBP-1, IGFBP-2, and IGFBP-3 and levels of each peptide were correlated with birth weight (BW), ponderal index (PI), and placental weight (PW). IGF-2 failed to correlate with any growth parameter. However, IGFBP-3 was associated significantly with BW, PI, PW and also correlated positively to IGF-1 levels. These data suggest an important role for IGF-1 and IGFBP-3 in regulating fetal growth at term, as well as a coordinated regulation during the later stage of gestation (Osorio et al., 1996). In a case report of a patient with PS and asymmetric hypertrophy of various parts of the body, serum levels of IGF-1 and especially IGF-2 and their major growth hormone dependent binding protein (IGFBP-3) were significantly reduced, although growth hormone secretion after a pharmacological stimulus was normal. In addition, imbalanced levels of specific IGFBPs in the affected tissues could be observed (Rudolph et al., 1994). Similar to these observations, patients with typical SOTOS show low plasma IGF-2, IGFBP-3, IGFBP-4, and increased proteolysis of IGFBP-3 in serum. However, no correlation with the pathophysiology of Sotos syndrome could be established (de Boer et al., 2004).

Analyses of a mouse-model for Weaver syndrome (wv) suggest that IGF-1 serum levels play an important role in postnatal growth during and after neurodegeneration of wv mice and that IGF-1’s regulation of systemic growth during and after puberty is likely modulated by androgen in male wv mice. In this wv model an increase in the levels of circulating IGF-1 and hepatic mRNA preceded the catch-up of body weight of pubertal male mice (Yao et al., 2005). It remains to be seen to what extent these data can be transferred to humans.

### 4.3 Molecular diagnosis

The dynamic development of *omic* technologies and their implementation in routine diagnostics is revolutionizing genetic testing. These assays allow comprehensive insights in the pathophysiology of genomic alterations, and the identified pathogenic variants are increasingly targeted in personalized treatment regiments.

However, several of the *omic* approaches (e.g., long-read technologies) are currently under development or applied in research and clinical study settings (Figure 2) (Smirnov et al., 2023).

In respect to diagnostic testing, it is generally recommended to introduce techniques like WES and WGS in a diagnostic setting only when it provides relevant improvements on diagnostic yield (Matthijs et al., 2016; Souche et al., 2022). Furthermore, only genes with a clear disease-association should be addressed diagnostically, other genes should be analyzed in research settings (Souche et al., 2022).

A stepwise diagnostic approach might be considered in disorders with (frequent) disease-specific alterations, as WES/WGS are time-consuming and expensive, and harbor the risk to identify unsolicited findings. An example for a stepwise testing is BWSp, as more than 70% of patients with a typical phenotype exhibit genomic alterations in 11p15.5 which can easily be identified by a methylation-specific multiplex ligation-dependent probe amplification assay (MS-MLPA) and other PCR-based assays (Brioude et al., 2018).

However, the referring clinician and the laboratory running the assay have to be aware of the limitations of the applied assays, their informativeness and the time, which might be needed to get a result. For each of the disorders of interest, the spectrum of possible types of genomic variants has to be considered as they might require different methodological strategies.

#### 4.3.1 Pathogenic single nucleotide variants - SNVs

The majority of pathogenic genomic variants comprises single nucleotide variants (SNVs, substitutions, single basepair deletions/duplications, indels, etc.), and these can be addressed by sequencing technologies. In case specific genomic regions (e.g., in PS (Lindhurst et al., 2011)) or single genes should be addressed (e.g., *CDKN1C* in BWSp (Brioude et al., 2018)), Sanger sequencing might be a suitable tool.

However, nearly all overgrowth syndromes are genetically heterogeneous, therefore next-generation sequencing assays should be applied to decipher their genetic basis. In the past, targeted MPS gene-panels had been developed, which only address genes for which an association with overgrowth has been reported. However, an increasing number of studies show that in patients with heterogeneous phenotypes WES and WGS are more appropriate tools as they significantly increase the diagnostic yield (Meyer et al., 2017; Maron et al., 2023). Thus, these strategies should strongly be considered in diagnostic testing of patients with overgrowth, despite the increasing of variants of unknown significance which might hinder the data interpretation, and of unsolicited findings outside the scope of the test (Blackburn et al., 2015). However is the sequencing depth of routine WES/WGS often not high enough to pick up somatic variants that occur in a mosaic, such as in the *PIK3CA* (PROS, CS, CLOVES) gene. Here the use of high-depth MPS sequencing with specific gene panels or a Droplet Digital™ PCR (ddPCR™) for molecular diagnosis of the respective overgrowth syndromes is more appropriate.

#### 4.3.2 Pathogenic copy number variants–CNVs (structural variants)

Copy number variants (CNVs) comprise deletions and duplications of single exons, whole and multiple genes. They significantly contribute to the mutational spectrum, with more than 50% in some overgrowth disorders like Sotos syndrome. In case only the overgrowth-disease specific gene is affected, the patients commonly show the disease-specific phenotype. The clinical phenotype can be modified in case further genes are affected by the imbalance, in these cases the clinical diagnosis might be impeded because the phenotype might then be influenced by disturbed doses of more than one gene.

In fact, a broad spectrum of (semi)quantitative assays has been developed to discriminate between copy numbers. They differ in the number of targetable genomic sequences. Whereas quantitative Real-Time PCR or digital PCR assays address only single regions, Multiplex Ligation-dependent Probe Amplification (MLPA) tests can address several dozens of sequences. On the other side, comprehensive and high resolution quantitative data are obtained by microarrays and CNV-targeting MPS approaches.

However, the choice which method is appropriate to diagnose a specific disorder depends on the spectrum of known variants. In case only single exon CNVs have to be considered, the use of a single target assay might be suitable, whereas larger variants like in SOTOS require the use of whole genomic tests.

The methods which are currently used in routine genetic testing only address loss and gain of genetic material, but genomically balanced structural variants are currently not covered. However, long-read technologies and optical mapping are currently validated and implemented in diagnostic algorithms of rare diseases, and they will provide further information about the role of structural variants to the etiology of overgrowth syndromes.

#### 4.3.3 Episignatures and imprinting defects

Epigenetic modifications as a cause of a disorder or as a biomarker for an entity are well-known for the molecularly defined group of imprinting disorders. The latter share the same types of molecular changes resulting in the disturbed expression of imprinted genes, i.e., genes underlying a strict monoallelic parent-of-origin specific expression (for review: (Eggermann et al., 2023)). Clinically, growth disturbances are a major clinical sign of imprinting disorders, among them BWSp.

However, in the last years a growing number of studies has provided evidence for the correlation of specific episignatures with neurodevelopmental disorders, among them overgrowth syndromes (Mannens et al., 2022) (Table 1). In these disorders present biomarkers indicate a malfunction of the underlying genetic defect. Thus, the main application of episignature testing is the functional evaluation of genomic variants in the specific genes and classification of variants of unknown significance in these factors. In contrast to imprinting disorders where the molecular diagnostic testing addresses the disease-specific loci, episignatures are determined by running epigenome-wide assays (i.e., array, whole genome-wide bisulphite sequencing) (Mannens et al., 2022; Smirnov et al., 2023). The underlying disturbance affects genes which encode proteins of the epigenetic machinery, and for several disorders highly specific epigenetic patterns have been reported. Thereby episignatures can support the clinical diagnosis and contribute to the pathogeneity classification of genetic variants (SNVs and CNVs).

#### 4.3.4 Mosaicism

Cellular mosaicism is a particular observation in overgrowth syndromes (e.g., in BWSp and *PIK3CA*-related overgrowth disorders) (Baker et al., 2021; Mussa et al., 2022), as several of them exhibit overgrowth only of parts of the body or even organs. As a consequence, routine genetic testing often reveals normal results as is it is conducted in peripheral lymphocytes.

In case of a strong suspicion of an overgrowth, testing of another tissue should therefore be considered. Depending on the clinical diagnosis and the reason for testing, buccal swab or hair root DNA might be helpful as it is an easily drawn specimen. However, DNA quality from these tissues might not be suitable for different assays, therefore direct tissue sampling of overgrowth affected tissues through biopsy or during surgical measurements are preferred. Nevertheless, even this targeted approach has to consider the purity of the biopsy to achieve the highest sensitivity for low-level mosaic detection.

The diagnostic application of several methods or their combination has been suggested to increase the sensitivity for detection of low-mosaicism (e.g., digital PCR, quantitative PCR, massive parallel sequencing). However, the problem of extremely differing mosaic distribution in different tissues remains. As mentioned before, analysis of different tissues is therefore an option to address this issue, but in the future the use of liquid biopsies might circumvent the aforementioned limitations (see 6).

## 5 Current and future management of overgrowth

The management of the depicted overgrowth syndromes depends on the syndrome and usually is a treatment of individual clinical manifestations. Since these range from endocrinologic deficits like neonatal hypoglycemia or hyperinsulinism to orthopedic- and aesthetic-deficits to neurologic/developmental delays and cancer predisposition, a detailed paragraph covering all management aspects for the listed overgrowth syndromes would exceed the scope of this review. For a detailed reading on overgrowth management we therefore would like to refer to Expert consensus publications, where available (e.g., on BWSp (Brioude et al., 2018) or PROS (Douzgou et al., 2022)). Where Consensus statements are not available by now, e.g., due to the low number of studies on a specific syndrome, we would recommend the corresponding articles in GeneReviews (Mirzaa et al., 1993; Shuman et al., 1993), an international point-of-care resource covering diagnosis, management and genetic counseling.

Since most causative (epi-)genetic alterations for the overgrowth syndromes affect cellular growth signaling cascades that are also supportive for tumor-development (Figure 1), some new management aspects addressing these molecular pathways come from the field of oncology. An example is the pharmacological inhibitor of PIK3CA (alpelisib) which has been approved in oncology for the treatment of advanced breast cancer (Andre et al., 2019). Initial studies in a *Pik3ca* mutant mouse model (Delestre et al., 2021) have suggested that alpelisib might also be used to improve the clinical outcome of PROS patients, for whom no approved medical therapy exists. A recent study on two infants with PROS having life-threatening conditions showed that low-dose alpelisib treatment was associated with improvement in signs and symptoms, morphological lesions and vascular anomalies in the two patients without obvious adverse events during the study (Morin et al., 2022). Approaches like these pave the road for new treatment options utilizing established treatment options from other medical fields also for the treatment of rare overgrowth syndromes.

The dynamics of technological progress does not only influence the therapeutic management, but also the individual prediction of specific symptoms by AI-based tools. An example is the recently developed Deeplasia tool, a deep learning software for bone age assessment which can be used to predict the bone development in skeletal dysplasia (Rassmann et al., 2024).

## 6 Discussion/perspectives

### 6.1 Genomic assays currently used in diagnostic algorithms

The molecular diagnostic analyses encompass DNA-, epigenetic- and transcriptomic-analyses that all have been increasingly developed during the last years (compare Figure 2). They have progressed from a single disease-gene sequencing to whole genome/exome/transcriptome sequencing approaches. These *omic* strategies can confirm a clinical diagnosis with the use of a broad, rather unfocused approach, but thus also produce quite a number of unsolicited findings, or variants of unknown significance (VUS). AI-based integrated methods will help to reclassify VUS according to their relevance for disease and ultimately detect molecular signatures of the overgrowth syndromes. This could also help to uncover trans-acting factors, e.g., for epigenetic trans-regulations (Eggermann et al., 2022) or gene-networks, like the Imprinted-Gene-Network (IGN) (Varrault et al., 2006; Eggermann et al., 2021). The plethora of information in *omics* data can thus be used to bioinformatically gain deeper insight into relevant genomic background effects. Examples are mutations in the *EZH2* (Weaver syndrome) and *DNMT3A* (Tatton-Brown-Rahman syndrome) which cause overgrowth, but are also candidates for the Facioscapulohumeral Dystrophy (FSHD) with a wide heterogeneity of disease, complicating FSHD diagnosis and the genotype-phenotype correlation among patients and within families. A recent study applied Whole Exome Sequencing (WES) to investigate known and unknown genetic contributors that may be involved in FSHD and may represent potential disease modifiers. The WES data analysis suggests that different genes can contribute to disease heterogeneity in presence of a FSHD permissive (genetic) background (Strafella et al., 2023). Similar strategies could be used to analyze overgrowth syndromes with causative genetic mutations. A combined segregation analyses for genetic variants in family members together with clinical findings and a methylation analysis probably will help to establish a reliable genotype-phenotype correlation of the complex overgrowth-syndromes.

### 6.2 Use of longread-technologies for diagnostic purposes

Current strategies in the sequencing technologies move also from the classical sequencing-by-synthesis (Sanger-sequencing) with rather short reads towards long-read technologies (e.g., Oxford Nanopore and PacBio platforms), which are able to read DNA (and RNA) sequences over several kilobases (Figure 2). These techniques not only register the sequence, but due to their base-detection methods are also able to detect modifications (e.g., 5mC, 5hmC), thus integrating two *omics* levels (sequence and methylation). Long-range phasing enables allele specific variant detection which includes identification of parental specific methylation (Gigante et al., 2019). These approaches will significantly improve our functional understanding of the maintenance of genomic imprinting and how associated disease phenotypes develop (e.g., BWSp) (Hubert and Demars, 2022). Last but not least, long-read assays allow the identification and characterization of structural chromosomal variants.

### 6.3 Future use of further *omic* technologies

Next-generation-based DNA sequencing strategies are already established in the clinical practice of rare diseases including overgrowth syndromes (Pagnamenta et al., 2023), however gene expression data are hardly utilized in routine clinical practice. A recent exploratory study has demonstrated the potential for gene expression profiling to aid in both, the diagnosis and classification of a disease entity. In this study, the authors examined whether gene expression profiling could distinguish children from the PREDICT study with growth hormone deficiency from healthy controls. This work illustrates how a single blood test with extracted mRNA applied to massive parallel sequencing (MPS) transcriptomic-analyses in combination with a machine learning algorithm in the future could replace, e.g., endocrine stimulation tests (Murray et al., 2018). The use of direct sequencing methods compatible with long reads will also be useful in the transcriptomics field (Figure 2). So does current data suggest that the regulation of gene expression is provided in part by long noncoding RNAs (lncRNAs), that have no coding potential, regulate genes *in cis* via transcriptional interference and to a certain extend seem to be integrated into riboprotein complexes that mediate epigenetic modifications in predetermined genomic regions (e.g., *H19*, a candidate for BWSp aspects) (Li et al., 2023). As these lncRNAs may be larger than one kilobase, long read direct RNA-seq methods seem to be an appropriate approach.

In the field of proteomics current strategies analyse the links between nuclear architecture, chromatin topology and genetic elements by utilizing Chromosome Conformation Capture (3C)-based technologies (C-technologies) (Rao et al., 2014) (Figure 2). Such strategies showed, for example, the enrichment of imprinted genes (e.g., causative for BWSp) in chromatin loops and suggest that subtle changes in contact propensity affect gene regulation and could be a mechanism by which regulatory genetic variants in chromatin loop anchors mediate effects on gene expression (Greenwald et al., 2019). Future studies will quantitatively examine how changes in chromatin loop formations elicit changes in expression (or *vice versa*), thus also help to determine how VUS, e.g., in loop anchor sequences, can influence gene expression and ultimately result in the phenotype. In the field of proteomics the Nanopore technology that has already been established for long read DNA-/RNA-sequencing can be used to sequence proteins (Motone et al., 2023) (Figure 2). This technique can be used to sequence single protein molecules in their native, full-length form and will enable a more comprehensive proteome analysis other than the classic Mass-Spec analyses. The relevant cellular signaling cascades (Figure 1), do influence the growth signals dependent on the quantity and effectivity of the cascade components, but also due to their post-translational modifications like the grade of phosphorylation. Consequently, the phosphoproteome, like already the metabolome, of patient cells will get more and more into the focus of analyses. The sensitivity of such proteomic analyses aims to reach single cell resolution like the nucleic-acid analyses mentioned above. In addition to the promising Nanopore technique recently trapped ion mobility spectrometry (TIMS) coupled to time of flight (TOF) analyzers have been established for such an ultra-high sensitivity proteomic analysis (Rosenberger et al., 2023) (Figure 2).

Current *omics* discussions also address the principal concept of a biological study. Most *omics* analyses use, for example, DNA or RNA extracted from a blood sample or cell culture as source for the data generation. These results are however quite heterogenous. So have most overgrowth syndromes that present with confined overgrowth of one or a few regions of the body (e.g., BWSp Hemihyperplasia, PHTS, PS, PIK3CA, PWS, KTS, PKWS and *Proteus* syndrome) the molecular defects in only a subset of cells (i.e., a mosaic distribution of the mutation). The generated *omics* data reflects the heterogeneity of the sample, with the causative alterations being only more or less obvious due to the degree of mosaicism. In addition, the cellular expression profiles generated by such a sample are also *per se* heterogeneous, because expression profiles change dynamically with their chronological or metabolic cellular conditions. This is a major caveat for the correct interpretation of almost all *omics* data (Yamada et al., 2021) and is the principal motivation of single-cell studies (Stuart and Satija, 2019) (Figure 2). With improving protocols single-cell studies will be utilized more often to obtain a less mixed data set.

The combination of different *omics* approaches paired with bioinformatics/Ai supports the identification of new Biomarkers also in the disease entities associated with the overgrowth syndromes (Figure 2), like endocrinologic complications or tumor development. So does fetal overgrowth predispose to type 2 diabetes (T2D) in adulthood. A recent study analyzed genome-wide alterations in placental gene methylations in fetal overgrowth and associated them with metabolic health biomarkers including leptin, adiponectin and fetal growth factors. The data suggest that fetal overgrowth appears to be associated with hypermethylation of *CDH13* and *VSX1* genes, which may be promising epigenetic biomarkers for an elevated risk of metabolic dysfunctional disorders in later life (Yang et al., 2022). Multi-omics approaches also provide an opportunity to stratify T2D patients into endotypes, thus improving therapeutic treatment and outcome. Among the *omics* analyses, epigenetics and metabolomics are gaining increasing interest (Figure 2). Recent studies show a dynamic correlation between metabolic pathways and gene expression through chromatin remodeling (e.g., via DNA methylation), thereby providing insight into altered physiological processes early in the endothelium that predispose to cardiovascular disease (CVD), the leading cause of morbidity and mortality in T2D patients disease development. The early phase of endothelial dysfunction provides starting points to identify disease biomarkers, as well as new therapies for CVD prevention (Di Pietrantonio et al., 2023).

As already mentioned, the main molecular players causative for the depicted overgrowth entities roughly represent gatekeepers for cellular growth. Altered cellular growth is also one of the main driving forces for tumor development. So does GPC-3 act as a cell cycle regulator. Loss-of-function mutations in this gene cause the SGBS. Proteomic and Transcriptomic studies in a number of cancer types have displayed an upregulation of GPC-3 expression, suggesting GPC-3 to be a useful marker for hepatocellular carcinoma (HCC). Since GPC-3 is a transmembrane heparan sulfate proteoglycan also an immunotherapy targeting this protein or affiliated proteins is under investigation in HCC and other diseases in which GPC-3 is overexpressed (Montalbano et al., 2017).

In respect to mosaicism as a major challenge in genetic diagnosis of several overgrowth syndromes, liquid biopsy might contribute to overcome the limitations of identifying somatic pathogenic variants above the background of the wildtype allele. Liquid biopsy analysis is based on MPS approaches of serum samples, and it has a high sensitivity to identify circulating cell free DNA (cfDNA) from mutant cells. Thereby, somatic variants can be identified, as the fraction of somatic mutant DNA can be identified due to the lethality of the mutant cell-type. In fact, the suitability of liquid biopsy to molecularly confirm mosaic overgrowth disorders in blood samples has been illustrated for PROS entities (Serio et al., 2022).

### 6.4 Data re-evaluation

To diagnose patients the *omics* techniques use the current knowledge and public databases to estimate the effect of identified alterations. However, as already discussed above, identified alterations (e.g., VUS) can also have an unclear significance for the clinical situation. The diagnostic analysis of *omics* data therefore may be limited by the available knowledge at the current time, as well as by the employed algorithms and filtering strategies. As explained in section 6.3 future studies will probably help to determine how VUS, e.g., in loop anchor sequences, can influence gene expression and ultimately result in the phenotype, thus adding functional aspects to the current VUS. Consequently reanalyzing *omics* data from patient cohorts will allow the identification of potentially missed or misclassified genetic variants and provide an opportunity to evaluate diagnostic variants according to current standards and guidelines (Bayat, 2023) (Figure 2). Manual re-analysis is time-consuming, therefore a computational workflow with optional filtering (e.g., re-analyses only of likely or known loss-of-function variants in ClinVar/HGMD) needs to be established to get the re-analysis into routine use. Re-analyses of apparently solved cases is also recommendable to identify over-classified variants and associations. A re-evaluation can be performed after certain time intervals (e.g., every 5 years as suggested by (Bartolomaeus et al., 2023)) but it is difficult to estimate what timeframes are adequate for which disease entity and if the re-analyses should be performed for all patients available, or only for those where certain levels of analysis have been performed already. In the light of the rapidly developing fields of *omic* techniques it is also worth considering if re-analyses should be performed with the old sequencing data sets or with new data generated by up-to-date techniques.

## 7 Summary

Overgrowth (macrosomia, tall stature) is defined as a body length that is above the 97th percentile of the reference population. Depending on the underlying physiological pathway affected by pathogenic genetic alterations, overgrowth syndromes are associated with a broad spectrum of neoplasia predisposition, (cardio) vascular and neurodevelopmental anomalies, and dysmorphisms (Table 1). Clinical features of overgrowth syndromes often overlap, impeding the clinical diagnosis of the disorders. The precise molecular diagnosis is necessary as the basis for an appropriate, disease-specific health issues. One major effector for somatic growth is the IGF1R-pathway, that plays a critical role in cell growth and differentiation by activating the PI3K-AKT-mTOR and RAS-RAF-mitogen-activated protein kinase (MAPK) pathways (Figure 1). Some of the overgrowth syndromes present with confined overgrowth of one or a few regions of the body (i.e., a mosaic distribution of the causative genetic variant). Epigenetic modifications (i.e., DNA methylation, histone modification) also are important for the manifestation of the rare diseases and specific episignatures have been reported (Table 1). The diagnosis of overgrowth syndromes often is challenging due to the clinical and molecular heterogeneities as well as subtle-, developing- and overlapping features. Biochemical diagnosis of overgrowth entities with focus on IGF-1, IGFBP-3, free T4, and TSH only represent an auxiliary evaluation test in addition to growth velocity analyses, karyotype studies and bone age.

The development of *omic* technologies and their implementation in routine diagnostics currently revolutionizes genetic testing (Figure 2) and increasingly can be used for personalized treatment regiments. Causative molecular alterations detected by *omics* technologies encompass mutations, SNV, CNV and epigenetic modifications. The combination of different *omics* approaches paired with bioinformatics/Ai (Figure 2) supports the identification of new Biomarkers and significantly improves our functional understanding of the analyzed disease phenotypes and give indications for the development of therapeutic agents. Mosaicism still is s a major challenge in genetic diagnosis of several overgrowth syndromes. Here liquid biopsy strategies and single cell analyses might contribute to overcome the limitations of identifying somatic pathogenic variants. Here will long-read sequencing techniques like the Nanopore systems (Figure 2) help to detect low-complexity repeat variants or specific structural variants missed by traditional exome/genome sequencing as well as enable the exploration of the non-coding genome including disruption of topologically associated domains, long range non-coding RNA, or other regulatory elements. These techniques are enabling a multi-*omics* approach by simultaneously or sequentially analyzing epigenetic signatures combined with the phased primary sequence. Transcriptomics and metabolomics as complementary diagnostic methods may be used in the assessment of variants of unknown significance. Some of these tools are currently not integrated into standard diagnostic workup. However, it is reasonable to expect that they will become increasingly available and improve current diagnostic capabilities, thereby enabling precision diagnosis in patients who are currently undiagnosed. Finally, a regular re-analysis of older exome/genome data will update and increase the knowledge of molecular effectors and over time increase the diagnostic yield (Figure 2).

## Abbreviations

3C, Chromosome Conformation Capture; 4C: 3C on Chip; 5C, 3C-Carbon Copy; ATAC-Seq, Assay for Transposase-Accessible Chromatin with high-throughput Sequencing; ChIA-PET, Chromatin Interaction Analysis with Paired-End Tag sequencing; ChIP-Seq, Chromatin ImmunoPrecipitation followed by Sequencing; DNAse-seq, DNase I hypersensitive sites sequencing; HiC, High-throughput 3C; IP, Immunoprecipitation; MLPA, multiplex ligation-dependent probe amplification; MS-based methods, Mass spectrometry based methods; MS-MLPA, methylation specific multiplex ligation-dependent probe amplification; MS-PCR, methylation specific polymerase chain reaction; Sc, single cell - (e.g., scRNA-Seq: single-cell RNA-Seq); sci-MET, single-cell combinatorial indexing for methylation analysis; snmC-seq3, single-nucleus methylome sequencing; TIMS, trapped ion mobility spectrometry; timsTOF, trapped ion mobility spectrometry (TIMS) coupled to time of flight (TOF) analyzers; WES, Whole-Exome Sequencing; WGS, Whole-Genome Sequencing; WG(B)S, Whole-Genome Bisulfite Sequencing.

## References

[B1] Alves Junior C. A. S. Martins P. C. Moreno Aznar L. A. Silva D. A. S. (2023). Reference growth curves to identify weight status (underweight, overweight or obesity) in children and adolescents: systematic review. Br. J. Nutr. 130 (4), 666–678. 10.1017/S0007114522003786 36695353

[B2] Andre F. Ciruelos E. Rubovszky G. Campone M. Loibl S. Rugo H. S. (2019). Alpelisib for PIK3CA-mutated, hormone receptor-positive advanced breast cancer. N. Engl. J. Med. 380 (20), 1929–1940. 10.1056/NEJMoa1813904 31091374

[B3] Baker S. W. Duffy K. A. Richards-Yutz J. Deardorff M. A. Kalish J. M. Ganguly A. (2021). Improved molecular detection of mosaicism in Beckwith-Wiedemann Syndrome. J. Med. Genet. 58 (3), 178–184. 10.1136/jmedgenet-2019-106498 32430359 PMC7959163

[B4] Baldwin A. Pirisi L. Creek K. E. (2004). NFI-Ski interactions mediate transforming growth factor beta modulation of human papillomavirus type 16 early gene expression. J. Virol. 78 (8), 3953–3964. 10.1128/jvi.78.8.3953-3964.2004 15047811 PMC374275

[B5] Bartolomaeus T. Hentschel J. Jamra R. A. Popp B. (2023). Re-evaluation and re-analysis of 152 research exomes five years after the initial report reveals clinically relevant changes in 18. Eur. J. Hum. Genet. 31 (10), 1154–1164. 10.1038/s41431-023-01425-6 37460657 PMC10545662

[B6] Bayat A. (2023). Unveiling the hidden: revisiting the potential of old genetic data for rare disease research. Eur. J. Hum. Genet. 31 (10), 1093–1094. 10.1038/s41431-023-01435-4 37474787 PMC10545786

[B7] Bayrak-Toydemir P. Stevenson D. A. (1993). “Capillary malformation-arteriovenous malformation syndrome,” in GeneReviews((R)). Editors Adam M. P. Feldman J. Mirzaa G. M. Pagon R. A. Wallace S. E. Bean L. J. H. (Washington: Seattle WA). 21348050

[B8] Biesecker L. G. Sapp J. C. (1993). “Proteus syndrome,” in GeneReviews((R)). Editors Adam M. P. Feldman J. Mirzaa G. M. Pagon R. A. Wallace S. E. Bean L. J. H. (Washington: Seattle WA). 22876373

[B9] Binder G. Eggermann T. Weber K. Ferrand N. Schweizer R. (2017). The diagnostic value of IGF-2 and the IGF/IGFBP-3 system in silver-russell syndrome. Horm. Res. Paediatr. 88 (3-4), 201–207. 10.1159/000477666 28675902

[B10] Binder G. Seidel A. K. Weber K. Haase M. Wollmann H. A. Ranke M. B. (2006). IGF-II serum levels are normal in children with Silver-Russell syndrome who frequently carry epimutations at the IGF2 locus. J. Clin. Endocrinol. Metab. 91 (11), 4709–4712. 10.1210/jc.2006-1127 16940449

[B11] Blackburn H. L. Schroeder B. Turner C. Shriver C. D. Ellsworth D. L. Ellsworth R. E. (2015). Management of incidental findings in the era of next-generation sequencing. Curr. Genomics 16 (3), 159–174. 10.2174/1389202916666150317232930 26069456 PMC4460220

[B12] Bracken A. P. Dietrich N. Pasini D. Hansen K. H. Helin K. (2006). Genome-wide mapping of Polycomb target genes unravels their roles in cell fate transitions. Genes Dev. 20 (9), 1123–1136. 10.1101/gad.381706 16618801 PMC1472472

[B13] Brioude F. Kalish J. M. Mussa A. Foster A. C. Bliek J. Ferrero G. B. (2018). Expert consensus document: clinical and molecular diagnosis, screening and management of Beckwith-Wiedemann syndrome: an international consensus statement. Nat. Rev. Endocrinol. 14 (4), 229–249. 10.1038/nrendo.2017.166 29377879 PMC6022848

[B14] Brioude F. Toutain A. Giabicani E. Cottereau E. Cormier-Daire V. Netchine I. (2019). Overgrowth syndromes - clinical and molecular aspects and tumour risk. Nat. Rev. Endocrinol. 15 (5), 299–311. 10.1038/s41574-019-0180-z 30842651

[B15] Burkardt D. Tatton-Brown K. (1993). “HIST1H1E syndrome,” in GeneReviews((R)). Editors Adam M. P. Feldman J. Mirzaa G. M. Pagon R. A. Wallace S. E. Bean L. J. H. (Washington: Seattle WA). 33270410

[B16] Cai X. Cullen B. R. (2007). The imprinted H19 noncoding RNA is a primary microRNA precursor. RNA 13 (3), 313–316. 10.1261/rna.351707 17237358 PMC1800509

[B17] Candela M. E. Cantley L. Yasuaha R. Iwamoto M. Pacifici M. Enomoto-Iwamoto M. (2014). Distribution of slow-cycling cells in epiphyseal cartilage and requirement of β-catenin signaling for their maintenance in growth plate. J. Orthop. Res. 32 (5), 661–668. 10.1002/jor.22583 24415663 PMC4114074

[B18] Capurro M. I. Li F. Filmus J. (2009). Overgrowth of a mouse model of Simpson-Golabi-Behmel syndrome is partly mediated by Indian hedgehog. EMBO Rep. 10 (8), 901–907. 10.1038/embor.2009.98 19590577 PMC2726674

[B19] Cha T. L. Zhou B. P. Xia W. Wu Y. Yang C. C. Chen C. T. (2005). Akt-mediated phosphorylation of EZH2 suppresses methylation of lysine 27 in histone H3. Science 310 (5746), 306–310. 10.1126/science.1118947 16224021

[B20] Cheng W. Tseng C. J. Lin T. T. Cheng I. Pan H. W. Hsu H. C. (2008). Glypican-3-mediated oncogenesis involves the Insulin-like growth factor-signaling pathway. Carcinogenesis 29 (7), 1319–1326. 10.1093/carcin/bgn091 18413366 PMC2500215

[B21] Chitnis M. M. Yuen J. S. Protheroe A. S. Pollak M. Macaulay V. M. (2008). The type 1 insulin-like growth factor receptor pathway. Clin. Cancer Res. 14 (20), 6364–6370. 10.1158/1078-0432.CCR-07-4879 18927274

[B22] Cyrus S. Burkardt D. Weaver D. D. Gibson W. T. (2019). PRC2-complex related dysfunction in overgrowth syndromes: a review of EZH2, EED, and SUZ12 and their syndromic phenotypes. Am. J. Med. Genet. C Semin. Med. Genet. 181 (4), 519–531. 10.1002/ajmg.c.31754 31724824

[B23] Damhuis S. E. Ganzevoort W. Gordijn S. J. (2021). Abnormal fetal growth: small for gestational age, fetal growth restriction, large for gestational age: definitions and epidemiology. Obstet. Gynecol. Clin. North Am. 48 (2), 267–279. 10.1016/j.ogc.2021.02.002 33972065

[B24] de Boer L. Hoogerbrugge C. M. van Doorn J. van Buul-Offers S. C. Karperien M. Wit J. M. (2004). Plasma insulin-like growth factors (IGFs), IGF-Binding proteins (IGFBPs), acid-labile subunit (ALS) and IGFBP-3 proteolysis in individuals with clinical characteristics of Sotos syndrome. J. Pediatr. Endocrinol. Metab. 17 (4), 615–627. 10.1515/jpem.2004.17.4.615 15198293

[B25] Delestre F. Venot Q. Bayard C. Fraissenon A. Ladraa S. Hoguin C. (2021). Alpelisib administration reduced lymphatic malformations in a mouse model and in patients. Sci. Transl. Med. 13 (614), eabg0809. 10.1126/scitranslmed.abg0809 34613809

[B26] Diaz Bonilla E. Torres Galvis C. L. Gomez Campos R. de Arruda M. Pacheco Carrillo J. Cossio Bolanos M. (2018). Weight, height and body mass index of children and adolescents living at moderate altitude in Colombia. Arch. Argent. Pediatr. 116 (2), e241–e250. 10.5546/aap.2018.eng.e241 29557607

[B27] Di Pietrantonio N. Cappellacci I. Mandatori D. Baldassarre M. P. A. Pandolfi A. Pipino C. (2023). Role of epigenetics and metabolomics in predicting endothelial dysfunction in type 2 diabetes. Adv. Biol. (Weinh) 7 (9), e2300172. 10.1002/adbi.202300172 37616517

[B28] Douzgou S. Janssens V. Houge G. (1993). “PPP2R1A-Related neurodevelopmental disorder,” in GeneReviews((R)). Editors Adam M. P. Feldman J. Mirzaa G. M. Pagon R. A. Wallace S. E. Bean L. J. H. (Washington: Seattle WA). 35593790

[B29] Douzgou S. Rawson M. Baselga E. Danielpour M. Faivre L. Kashanian A. (2022). A standard of care for individuals with PIK3CA-related disorders: an international expert consensus statement. Clin. Genet. 101 (1), 32–47. 10.1111/cge.14027 34240408 PMC8664971

[B30] Duzenli T. Sezer A. Kayhan G. Arslan A. T. Percin F. E. (2023). Expanding the phenotype and genotype in Thauvin-Robinet-Faivre syndrome: a new patient with a novel variant and additional clinical findings. Am. J. Med. Genet. A 191 (8), 2232–2239. 10.1002/ajmg.a.63300 37218527

[B31] Eggermann T. Davies J. H. Tauber M. van den Akker E. Hokken-Koelega A. Johansson G. (2021). Growth restriction and genomic imprinting-overlapping phenotypes support the concept of an imprinting network. Genes (Basel) 12 (4), 585. 10.3390/genes12040585 33920525 PMC8073901

[B32] Eggermann T. Monk D. de Nanclares G. P. Kagami M. Giabicani E. Riccio A. (2023). Imprinting disorders. Nat. Rev. Dis. Prim. 9 (1), 33. 10.1038/s41572-023-00443-4 37386011

[B33] Eggermann T. Yapici E. Bliek J. Pereda A. Begemann M. Russo S. (2022). Trans-acting genetic variants causing multilocus imprinting disturbance (MLID): common mechanisms and consequences. Clin. Epigenetics 14 (1), 41. 10.1186/s13148-022-01259-x 35296332 PMC8928698

[B34] Gigante S. Gouil Q. Lucattini A. Keniry A. Beck T. Tinning M. (2019). Using long-read sequencing to detect imprinted DNA methylation. Nucleic Acids Res. 47 (8), e46. 10.1093/nar/gkz107 30793194 PMC6486641

[B35] Grange D. K. Nichols C. G. Singh G. K. (1993). “Cantu syndrome,” in GeneReviews((R)). Editors Adam M. P. Feldman J. Mirzaa G. M. Pagon R. A. Wallace S. E. Bean L. J. H. (Washington: Seattle WA). 25275207

[B36] Greenwald W. W. Li H. Benaglio P. Jakubosky D. Matsui H. Schmitt A. (2019). Subtle changes in chromatin loop contact propensity are associated with differential gene regulation and expression. Nat. Commun. 10 (1), 1054. 10.1038/s41467-019-08940-5 30837461 PMC6401380

[B37] Greysson-Wong J. Rode R. Ryu J. R. Chan J. L. Davari P. Rinker K. D. (2023). rasa1-related arteriovenous malformation is driven by aberrant venous signalling. Development 150 (18), dev201820. 10.1242/dev.201820 37708300

[B38] Guihard-Costa A. M. Droulle P. Larroche J. C. (1991). Growth velocity of the biparietal diameter, abdominal transverse diameter and femur length in the fetal period. Early Hum. Dev. 27 (1-2), 93–102. 10.1016/0378-3782(91)90030-7 1802668

[B39] Gurovich Y. Hanani Y. Bar O. Nadav G. Fleischer N. Gelbman D. (2019). Identifying facial phenotypes of genetic disorders using deep learning. Nat. Med. 25 (1), 60–64. 10.1038/s41591-018-0279-0 30617323

[B40] Hallett S. A. Ono W. Ono N. (2021). The hypertrophic chondrocyte: to be or not to be. Histol. Histopathol. 36 (10), 1021–1036. 10.14670/HH-18-355 34137454 PMC8678381

[B41] Hsieh T. C. Mensah M. A. Pantel J. T. Aguilar D. Bar O. Bayat A. (2019). PEDIA: prioritization of exome data by image analysis. Genet. Med. 21 (12), 2807–2814. 10.1038/s41436-019-0566-2 31164752 PMC6892739

[B42] Hubert J. N. Demars J. (2022). Genomic imprinting in the new omics era: a model for systems-level approaches. Front. Genet. 13, 838534. 10.3389/fgene.2022.838534 35368671 PMC8965095

[B43] Inada M. Wang Y. Byrne M. H. Rahman M. U. Miyaura C. Lopez-Otin C. (2004). Critical roles for collagenase-3 (Mmp13) in development of growth plate cartilage and in endochondral ossification. Proc. Natl. Acad. Sci. U. S. A. 101 (49), 17192–17197. 10.1073/pnas.0407788101 15563592 PMC535367

[B44] Kanaan R. A. Kanaan L. A. (2006). Transforming growth factor beta1, bone connection. Med. Sci. Monit. 12 (8), RA164–9. 16865078

[B45] Karsenty G. (2003). The complexities of skeletal biology. Nature 423 (6937), 316–318. 10.1038/nature01654 12748648

[B46] Klein S. D. Nisbet A. F. Hathaway E. R. Kalish J. M. (1993). “Simpson-golabi-behmel syndrome type 1,” in GeneReviews((R)). Editors Adam M. P. Feldman J. Mirzaa G. M. Pagon R. A. Wallace S. E. Bean L. J. H. (Washington: Seattle WA). 20301398

[B47] Kohler S. Schulz M. H. Krawitz P. Bauer S. Dolken S. Ott C. E. (2009). Clinical diagnostics in human genetics with semantic similarity searches in ontologies. Am. J. Hum. Genet. 85 (4), 457–464. 10.1016/j.ajhg.2009.09.003 19800049 PMC2756558

[B48] Kohler S. Vasilevsky N. A. Engelstad M. Foster E. McMurry J. Ayme S. (2017). The human phenotype Ontology in 2017. Nucleic Acids Res. 45 (D1), D865–D76. 10.1093/nar/gkw1039 27899602 PMC5210535

[B49] Kovacs C. S. (2014). Bone metabolism in the fetus and neonate. Pediatr. Nephrol. 29 (5), 793–803. 10.1007/s00467-013-2461-4 23529641

[B50] Kronenberg H. M. (2003). Developmental regulation of the growth plate. Nature 423 (6937), 332–336. 10.1038/nature01657 12748651

[B51] Kullmann M. K. Pegka F. Ploner C. Hengst L. (2021). Stimulation of c-jun/AP-1-activity by the cell cycle inhibitor p57(kip2). Front. Cell Dev. Biol. 9, 664609. 10.3389/fcell.2021.664609 33928088 PMC8076676

[B52] Li X. J. Zhou F. Li Y. J. Xue X. Y. Qu J. R. Fan G. F. (2023). LncRNA H19-EZH2 interaction promotes liver fibrosis via reprogramming H3K27me3 profiles. Acta Pharmacol. Sin. 44 (12), 2479–2491. 10.1038/s41401-023-01145-z 37580495 PMC10692088

[B53] Lindhurst M. J. Sapp J. C. Teer J. K. Johnston J. J. Finn E. M. Peters K. (2011). A mosaic activating mutation in AKT1 associated with the Proteus syndrome. N. Engl. J. Med. 365 (7), 611–619. 10.1056/NEJMoa1104017 21793738 PMC3170413

[B54] Lui J. C. Baron J. (2023). Epigenetic causes of overgrowth syndromes. J. Clin. Endocrinol. Metab. 109, 312–320. 10.1210/clinem/dgad420 PMC11032252 37450557

[B55] Mannens M. Lombardi M. P. Alders M. Henneman P. Bliek J. (2022). Further introduction of DNA methylation (DNAm) arrays in regular diagnostics. Front. Genet. 13, 831452. 10.3389/fgene.2022.831452 35860466 PMC9289263

[B56] Manor J. Lalani S. R. (2020). Overgrowth syndromes-evaluation, diagnosis, and management. Front. Pediatr. 8, 574857. 10.3389/fped.2020.574857 33194904 PMC7661798

[B57] Maron J. L. Kingsmore S. Gelb B. D. Vockley J. Wigby K. Bragg J. (2023). Rapid whole-genomic sequencing and a targeted neonatal gene panel in infants with a suspected genetic disorder. JAMA 330 (2), 161–169. 10.1001/jama.2023.9350 37432431 PMC10336625

[B58] Martinez-Delgado B. Barrero M. J. (2022). Epigenomic approaches for the diagnosis of rare diseases. Epigenomes 6 (3), 21. 10.3390/epigenomes6030021 35997367 PMC9397041

[B59] Matthijs G. Souche E. Alders M. Corveleyn A. Eck S. Feenstra I. (2016). Guidelines for diagnostic next-generation sequencing. Eur. J. Hum. Genet. 24 (1), 1515–5. 10.1038/ejhg.2016.63 PMC5027692 27628564

[B60] McClenaghan C. Nichols C. G. (2022). Kir6.1 and SUR2B in Cantu syndrome. Am. J. Physiol. Cell Physiol. 323 (3), C920–C935. 10.1152/ajpcell.00154.2022 35876283 PMC9467476

[B61] Menezes M. R. Balzeau J. Hagan J. P. (2018). 3' RNA uridylation in epitranscriptomics, gene regulation, and disease. Front. Mol. Biosci. 5, 61. 10.3389/fmolb.2018.00061 30057901 PMC6053540

[B62] Meyer R. Soellner L. Begemann M. Dicks S. Fekete G. Rahner N. (2017). Targeted next generation sequencing approach in patients referred for silver-russell syndrome testing increases the mutation detection rate and provides decisive information for clinical management. J. Pediatr. 187, 206–212. 10.1016/j.jpeds.2017.04.018 28529015

[B63] Mirzaa G. Graham J. M. Jr. Keppler-Noreuil K. (1993). “PIK3CA-Related overgrowth spectrum,” in GeneReviews((R)). Editors Adam M. P. Feldman J. Mirzaa G. M. Pagon R. A. Wallace S. E. Bean L. J. H. (Washington: Seattle WA). 23946963

[B64] Mitchel M. W. Myers S. M. Heidlebaugh A. R. Taylor C. M. Rea H. Neuhaus E. (1993). “CHD8-Related neurodevelopmental disorder with overgrowth,” in GeneReviews((R)). Editors Adam M. P. Feldman J. Mirzaa G. M. Pagon R. A. Wallace S. E. Bean L. J. H. (Seattle (WA). 36302072

[B65] Montalbano M. Georgiadis J. Masterson A. L. McGuire J. T. Prajapati J. Shirafkan A. (2017). Biology and function of glypican-3 as a candidate for early cancerous transformation of hepatocytes in hepatocellular carcinoma (Review). Oncol. Rep. 37 (3), 1291–1300. 10.3892/or.2017.5387 28098909

[B66] Montgomery N. D. Yee D. Chen A. Kalantry S. Chamberlain S. J. Otte A. P. (2005). The murine polycomb group protein Eed is required for global histone H3 lysine-27 methylation. Curr. Biol. 15 (10), 942–947. 10.1016/j.cub.2005.04.051 15916951

[B67] Morin G. Degrugillier-Chopinet C. Vincent M. Fraissenon A. Aubert H. Chapelle C. (2022). Treatment of two infants with PIK3CA-related overgrowth spectrum by alpelisib. J. Exp. Med. 219 (3), e20212148. 10.1084/jem.20212148 35080595 PMC8932545

[B68] Motone K. Kontogiorgos-Heintz D. Wee J. Kurihara K. Yang S. Roote G. (2023) Multi-pass, single-molecule nanopore reading of long protein strands with single-amino acid sensitivity. bioRxiv. 10.1038/s41586-024-07935-7 PMC11410661 39261738

[B69] Muench D. E. Ferchen K. Velu C. S. Pradhan K. Chetal K. Chen X. (2018). SKI controls MDS-associated chronic TGF-β signaling, aberrant splicing, and stem cell fitness. Blood 132 (21), e24–e34. 10.1182/blood-2018-06-860890 30249787 PMC6251005

[B70] Murray P. G. Stevens A. De Leonibus C. Koledova E. Chatelain P. Clayton P. E. (2018). Transcriptomics and machine learning predict diagnosis and severity of growth hormone deficiency. JCI Insight 3 (7), e93247. 10.1172/jci.insight.93247 29618660 PMC5928867

[B71] Mussa A. Leoni C. Iacoviello M. Carli D. Ranieri C. Pantaleo A. (2022). Genotypes and phenotypes heterogeneity in PIK3CA-related overgrowth spectrum and overlapping conditions: 150 novel patients and systematic review of 1007 patients with PIK3CA pathogenetic variants. J. Med. Genet. 60, 163–173. 10.1136/jmedgenet-2021-108093 35256403

[B72] Nahavandi S. Price S. Sumithran P. Ekinci E. I. (2019). Exploration of the shared pathophysiological mechanisms of gestational diabetes and large for gestational age offspring. World J. Diabetes 10 (6), 333–340. 10.4239/wjd.v10.i6.333 31231456 PMC6571486

[B73] Ornitz D. M. Marie P. J. (2015). Fibroblast growth factor signaling in skeletal development and disease. Genes Dev. 29 (14), 1463–1486. 10.1101/gad.266551.115 26220993 PMC4526732

[B74] Osorio M. Torres J. Moya F. Pezzullo J. Salafia C. Baxter R. (1996). Insulin-like growth factors (IGFs) and IGF binding proteins-1, -2, and -3 in newborn serum: relationships to fetoplacental growth at term. Early Hum. Dev. 46 (1-2), 15–26. 10.1016/0378-3782(96)01737-9 8899351

[B75] Ostrowski P. J. Tatton-Brown K. (1993). “Tatton-Brown-Rahman syndrome,” in GeneReviews((R)). Editors Adam M. P. Feldman J. Mirzaa G. M. Pagon R. A. Wallace S. E. Bean L. J. H. (Washington: Seattle WA). 35771960

[B76] Pagnamenta A. T. Camps C. Giacopuzzi E. Taylor J. M. Hashim M. Calpena E. (2023). Structural and non-coding variants increase the diagnostic yield of clinical whole genome sequencing for rare diseases. Genome Med. 15 (1), 94. 10.1186/s13073-023-01240-0 37946251 PMC10636885

[B77] Pappas J. Rabin R. (1993). “SETD2 neurodevelopmental disorders,” in GeneReviews((R)). Editors Adam M. P. Feldman J. Mirzaa G. M. Pagon R. A. Wallace S. E. Bean L. J. H. (Washington: Seattle WA). 34978780

[B78] Pedrazza L. Martinez-Martinez A. Sanchez-de-Diego C. Valer J. A. Pimenta-Lopes C. Sala-Gaston J. (2023). HERC1 deficiency causes osteopenia through transcriptional program dysregulation during bone remodeling. Cell Death Dis. 14 (1), 17. 10.1038/s41419-023-05549-x 36635269 PMC9837143

[B79] Qin X. Jiang Q. Nagano K. Moriishi T. Miyazaki T. Komori H. (2020). Runx2 is essential for the transdifferentiation of chondrocytes into osteoblasts. PLoS Genet. 16 (11), e1009169. 10.1371/journal.pgen.1009169 33253203 PMC7728394

[B80] Rao S. S. Huntley M. H. Durand N. C. Stamenova E. K. Bochkov I. D. Robinson J. T. (2014). A 3D map of the human genome at kilobase resolution reveals principles of chromatin looping. Cell 159 (7), 1665–1680. 10.1016/j.cell.2014.11.021 25497547 PMC5635824

[B81] Rassmann S. Keller A. Skaf K. Hustinx A. Gausche R. Ibarra-Arrelano M. A. (2024). Deeplasia: deep learning for bone age assessment validated on skeletal dysplasias. Pediatr. Radiol. 54 (1), 82–95. 10.1007/s00247-023-05789-1 37953411 PMC10776485

[B82] Rosenberger F. A. Thielert M. Mann M. (2023). Making single-cell proteomics biologically relevant. Nat. Methods 20 (3), 320–323. 10.1038/s41592-023-01771-9 36899157

[B83] Rudolph G. Blum W. F. Jenne E. W. Schoning M. Enders H. Meitinger T. (1994). Growth hormone (GH), insulin-like growth factors (IGFs), and IGF-binding protein-3 (IGFBP-3) in a child with Proteus syndrome. Am. J. Med. Genet. 50 (2), 204–210. 10.1002/ajmg.1320500213 7516626

[B84] Schultz K. A. P. Stewart D. R. Kamihara J. Bauer A. J. Merideth M. A. Stratton P. (1993). “DICER1 tumor predisposition,” in GeneReviews((R)). Editors Adam M. P. Feldman J. Mirzaa G. M. Pagon R. A. Wallace S. E. Bean L. J. H. (Washington: Seattle WA).

[B85] Semenza G. L. (2000). Expression of hypoxia-inducible factor 1: mechanisms and consequences. Biochem. Pharmacol. 59 (1), 47–53. 10.1016/s0006-2952(99)00292-0 10605934

[B86] Sequerra Amram Cohen A. Gibson W. T. (1993). “EED-related overgrowth,” in GeneReviews((R)). Editors Adam M. P. Feldman J. Mirzaa G. M. Pagon R. A. Wallace S. E. Bean L. J. H. (Washington: Seattle WA). 30973693

[B87] Serio V. B. Palmieri M. Loberti L. Granata S. Fallerini C. Vaghi M. (2022). Nosological and theranostic approach to vascular malformation through cfDNA NGS liquid biopsy. J. Clin. Med. 11 (13), 3740. 10.3390/jcm11133740 35807022 PMC9267326

[B88] Sferruzzi-Perri A. N. Sandovici I. Constancia M. Fowden A. L. (2017). Placental phenotype and the insulin-like growth factors: resource allocation to fetal growth. J. Physiol. 595 (15), 5057–5093. 10.1113/JP273330 28337745 PMC5538190

[B89] Shao R. Zhang Z. Xu Z. Ouyang H. Wang L. Ouyang H. (2021). H3K36 methyltransferase NSD1 regulates chondrocyte differentiation for skeletal development and fracture repair. Bone Res. 9 (1), 30. 10.1038/s41413-021-00148-y 34099628 PMC8185073

[B90] Shen G. (2005). The role of type X collagen in facilitating and regulating endochondral ossification of articular cartilage. Orthod. Craniofac Res. 8 (1), 11–17. 10.1111/j.1601-6343.2004.00308.x 15667640

[B91] Shen W. Flores-Daboub J. Viskochil D. Dugan S. L. Best H. D. Mao R. (2020). Detecting mosaic variants in patients with somatic overgrowth syndromes using cell-free circulating DNA and deep sequencing. J. Med. Genet. 57 (11), 794–796. 10.1136/jmedgenet-2019-106732 31992579

[B92] Shuman C. Kalish J. M. Weksberg R. (1993). “Beckwith-wiedemann syndrome,” in GeneReviews((R)). Editors Adam M. P. Feldman J. Mirzaa G. M. Pagon R. A. Wallace S. E. Bean L. J. H. (Washington: Seattle WA). 20301568

[B93] Smirnov D. Konstantinovskiy N. Prokisch H. (2023). Integrative omics approaches to advance rare disease diagnostics. J. Inherit. Metab. Dis. 46 (5), 824–838. 10.1002/jimd.12663 37553850

[B94] Smith A. M. LaValle T. A. Shinawi M. Ramakrishnan S. M. Abel H. J. Hill C. A. (2021). Functional and epigenetic phenotypes of humans and mice with DNMT3A Overgrowth Syndrome. Nat. Commun. 12 (1), 4549. 10.1038/s41467-021-24800-7 34315901 PMC8316576

[B95] Souche E. Beltran S. Brosens E. Belmont J. W. Fossum M. Riess O. (2022). Recommendations for whole genome sequencing in diagnostics for rare diseases. Eur. J. Hum. Genet. 30 (9), 1017–1021. 10.1038/s41431-022-01113-x 35577938 PMC9437083

[B96] Stevenson R. E. H. J. (2015) Human malformations and related anomalies. Third ed. New York: Oxford University Press.

[B97] Strafella C. Caputo V. Bortolani S. Torchia E. Megalizzi D. Trastulli G. (2023). Whole exome sequencing highlights rare variants in CTCF, DNMT1, DNMT3A, EZH2 and SUV39H1 as associated with FSHD. Front. Genet. 14, 1235589. 10.3389/fgene.2023.1235589 37674478 PMC10477786

[B98] Stuart T. Satija R. (2019). Integrative single-cell analysis. Nat. Rev. Genet. 20 (5), 257–272. 10.1038/s41576-019-0093-7 30696980

[B99] Takenouchi T. Okuno H. Kosaki K. (2019). Kosaki overgrowth syndrome: a newly identified entity caused by pathogenic variants in platelet-derived growth factor receptor-beta. Am. J. Med. Genet. C Semin. Med. Genet. 181 (4), 650–657. 10.1002/ajmg.c.31755 31710779

[B100] Tatton-Brown K. Cole T. R. P. Rahman N. (1993a). “Sotos syndrome,” in GeneReviews((R)). Editors Adam M. P. Feldman J. Mirzaa G. M. Pagon R. A. Wallace S. E. Bean L. J. H. (Washington: Seattle). 35771960

[B101] Tatton-Brown K. Rahman N. (1993b). “EZH2-Related overgrowth,” in GeneReviews((R)). Editors Adam M. P. Feldman J. Mirzaa G. M. Pagon R. A. Wallace S. E. Bean L. J. H. (Washington: Seattle WA). 23865096

[B102] Tenorio-Castano J. A. Arias P. Fernandez-Jaen A. Lay-Son G. Bueno-Lozano G. Bayat A. (2021). Tenorio syndrome: description of 14 novel cases and review of the clinical and molecular features. Clin. Genet. 100 (4), 405–411. 10.1111/cge.14020 34196401

[B103] Tsang K. Y. Chan D. Cheah K. S. (2015). Fate of growth plate hypertrophic chondrocytes: death or lineage extension? Dev. Growth Differ. 57 (2), 179–192. 10.1111/dgd.12203 25714187

[B104] Valcourt U. Ronziere M. C. Winkler P. Rosen V. Herbage D. Mallein-Gerin F. (1999). Different effects of bone morphogenetic proteins 2, 4, 12, and 13 on the expression of cartilage and bone markers in the MC615 chondrocyte cell line. Exp. Cell Res. 251 (2), 264–274. 10.1006/excr.1999.4584 10471312

[B105] Vallee A. Lecarpentier Y. Guillevin R. Vallee J. N. (2017). Interactions between TGF-β1, canonical WNT/β-catenin pathway and PPAR γ in radiation-induced fibrosis. Oncotarget 8 (52), 90579–90604. 10.18632/oncotarget.21234 29163854 PMC5685775

[B106] Varrault A. Gueydan C. Delalbre A. Bellmann A. Houssami S. Aknin C. (2006). Zac1 regulates an imprinted gene network critically involved in the control of embryonic growth. Dev. Cell 11 (5), 711–722. 10.1016/j.devcel.2006.09.003 17084362

[B107] Wang R. Zhou S. Wu P. Li M. Ding X. Sun L. (2018). Identifying involvement of H19-miR-675-3p-IGF1R and H19-miR-200a-PDCD4 in treating pulmonary hypertension with melatonin. Mol. Ther. Nucleic Acids 13, 44–54. 10.1016/j.omtn.2018.08.015 30240970 PMC6146608

[B108] Yamada R. Okada D. Wang J. Basak T. Koyama S. (2021). Interpretation of omics data analyses. J. Hum. Genet. 66 (1), 93–102. 10.1038/s10038-020-0763-5 32385339 PMC7728595

[B109] Yang M. N. Huang R. Zheng T. Dong Y. Wang W. J. Xu Y. J. (2022). Genome-wide placental DNA methylations in fetal overgrowth and associations with leptin, adiponectin and fetal growth factors. Clin. Epigenetics 14 (1), 192. 10.1186/s13148-022-01412-6 36585686 PMC9801645

[B110] Yao W. Zhong J. Rosen C. J. Hock J. M. Lee W. H. (2005). Igf-I and postnatal growth of weaver mutant mice. Endocrine 26 (2), 117–125. 10.1385/ENDO:26:2:117 15888923

[B111] Yehia L. Eng C. (1993). “PTEN hamartoma tumor syndrome,” in GeneReviews((R)). Editors Adam M. P. Feldman J. Mirzaa G. M. Pagon R. A. Wallace S. E. Bean L. J. H. (Washington: Seattle). 20301661

[B112] Zelzer E. Glotzer D. J. Hartmann C. Thomas D. Fukai N. Soker S. (2001). Tissue specific regulation of VEGF expression during bone development requires Cbfa1/Runx2. Mech. Dev. 106 (1-2), 97–106. 10.1016/s0925-4773(01)00428-2 11472838

[B113] Zhang Q. Qin S. Huai J. Yang H. Wei Y. (2023). Overexpression of IGF2 affects mouse weight and glycolipid metabolism and IGF2 is positively related to macrosomia. Front. Endocrinol. (Lausanne) 14, 1030453. 10.3389/fendo.2023.1030453 37152930 PMC10154688

[B114] Zhang S. Jiang M. Cao H. Xiong J. Xu J. (2022). CTB-193M12.5 promotes hepatocellular carcinoma progression via enhancing NSD1-mediated WNT10B/Wnt/β-Catenin signaling activation. J. Hepatocell. Carcinoma 9, 553–569. 10.2147/JHC.S365302 35698644 PMC9188405

